# Profile of Executive Functioning and Lifetime History of Acquired Brain Injury in Young Adults Experiencing Homelessness: A Pilot Study

**DOI:** 10.3390/ijerph22050790

**Published:** 2025-05-17

**Authors:** Nicole Viola, Julianna M. Nemeth, Alice Hinton, Jennifer P. Lundine

**Affiliations:** 1 Department of Speech and Hearing Science, The Ohio State University, Columbus, OH 43210, USA; lundine.4@osu.edu; 2 College of Public Health, The Ohio State University, Columbus, OH 43210, USA; nemeth.37@osu.edu; 3 Center for Tobacco Research, The Ohio State University, Columbus, OH 43210, USA; alice.hinton@osumc.edu; 4 Nationwide Children’s Hospital, Columbus, OH 43205, USA

**Keywords:** brain injuries, executive function, intervention, youth homelessness

## Abstract

(1) Background: Housing instability is a public health issue in both developed and developing countries. This exploratory pilot study examines executive functioning (EF) and the history of acquired brain injury (ABI) in youth and young adults experiencing homelessness (YYEH). (2) Methods: Twenty-three YYEH (ages 18–25; 48% male) were recruited from a drop-in center in Central Ohio. The Ohio State University TBI Identification Method (OSU TBI-ID+ABI) was used to screen for exposure to ABI. Outcomes were measured using the Behavior Rating Inventory of Executive Function for Adults (BRIEF-A) and the NIH Toolbox Cognitive Battery. (3) Results: Eighty-seven percent of participants reported a lifetime history of ABI, including traumatic brain injury or hypoxic-anoxic brain injury. Overall, participants did not present with elevated EF scores on outcome measures. Those with multiple lifetime ABIs (n = 16) had significantly higher scores than those with a single ABI (n = 4), representing poorer EF, on shift, self-monitor, task monitor, and the Global Executive Composite of the BRIEF-A. There was no evidence of significant differences between participants on outcome measures based on injury mechanism. (4) Conclusions: In this pilot sample, those with multiple reported ABIs demonstrated decreased EF performance, and these differences were noted in specific areas of EF. To ensure YYEH have access to services, resource support and intervention providers should consider the cognitive profiles of the youth they serve.

## 1. Introduction

Homelessness presents as a major public health concern worldwide. Due to differences in definitions and reporting metrics, it is difficult to definitively report the global prevalence of homelessness. However, it is estimated that more than 1.8 million people worldwide currently lack adequate housing [1]. In the United States, approximately 10% of youth and young adults aged 18 to 25 experience homelessness (YYEH) each year [2]. YYEH have historically reported higher exposure to adverse childhood events (ACEs), with 85.5% of unaccompanied YYEH reporting at least one ACE over their lifetime [3]. ACEs are defined as “potentially traumatic events that occur in childhood (0–17 years)” and include abuse and violence [4].With increased ACEs, the risk of experiencing an acquired brain injury (ABI) also increases [5]. ABIs may be traumatic or non-traumatic in origin. Traumatic brain injuries (TBIs) are caused by a bump or blow to the head via mechanisms such as a fall or being struck in the head. Non-traumatic injuries may be acquired from mechanisms such as stroke, infection, or deprivation of oxygen that can occur during cardiac arrest or strangulation. Nemeth et al. [6] found that in a sample of YYEH, a majority reported exposure to oxygen deprivation (i.e., choking games or intentional choking) or blunt force head trauma (i.e., being hit in the head or shaken violently). While the prevalence of ABI in YYEH has been understudied, it is estimated that 43–60% have a history of lifetime ABI [6,7]. This number is markedly higher than the estimated prevalence of 1.1% among youth and young adults in the general population [8].

Executive functioning (EF) is commonly impaired following ABI [9]. EFs are the skills that guide our intentional cognitive behavior and include three main categories: inhibitory control, working memory, and cognitive flexibility [10,11]. As such, when an individual experiences an ABI, they may demonstrate more impulsive behavior, problems with attention, or difficulty planning [12]. Impaired EF in YYEH may result in riskier decision-making and difficulty guiding goal-directed behaviors to aid in resource access for vulnerable youth, such as accessing drop-in centers or shelters, career counseling, and housing resources.

It is essential to consider the potential for EF difficulties and a prior history of ABI in YYEH. Currently, resource support and interventions may not consider the cognitive profiles of constituents served, despite the potential for vulnerable populations to experience a lifetime history of ABI and potentially associated EF deficits. Therefore, to better inform resources and supports, it is critical to build a profile of EF in YYEH who may or may not have a history of lifetime ABI. The goal of the current pilot study was to assess our ability to collect preliminary data on the following research questions: What types of ABI history are reported by YYEH? Do YYEH present with EF deficits? Is there a difference in the EF of YYEH based on the number and mechanism of ABI that they report throughout their lifetime? It was hypothesized that (1) there will be a combination of both traumatic and non-traumatic brain injury reported; (2) YYEH will present with elevated levels of executive dysfunction; (3) YYEH will demonstrate a difference in EF based on the number and mechanism of lifetime reported ABI.

## 2. Materials and Methods

### 2.1. Participants and Procedures

Institutional Review Board (IRB) approval for all study procedures was obtained prior to study initiation at The Ohio State University (#2023C0091). This study is a sub-analysis of cross-sectional data collected as part of a parent research study, Quitting Using Executive Strategy Training (QUEST). A primary aim of the QUEST project is to collect data needed to build an intervention to help YYEH (1) better access evidence-based smoking cessation resources that account for potential EF deficits; (2) adhere to processes of evidence-based treatment; and (3) address stress to prevent smoking relapse. Youth were eligible to participate in QUEST if they: (1) were 18–24 years old, (2) used combustible tobacco at least some days in the past week, and (3) were willing to stop smoking in the next 30 days. As this was part of a larger study focused on the use of a free smoking cessation resource, youth had to have access to a phone, enroll in a free online smoking cessation resource, share their quit line data with the study team, and provide biospecimens. Participants were ineligible if they did not have conversational English skills.

Participants were recruited at a drop-in center for YYEH in Central Ohio. The center provides YYEH immediate access to food, laundry, hygiene items, showers, and security. Additionally, the center connects youth to resources for counseling, legal aid, housing assistance, and more. Recruitment was conducted by trained graduate research assistants (RAs) who attended the drop-in center 5 days a week over 8 consecutive weeks and approached YYEH to assess their interest in study participation. To control for selection bias, this pilot study involved the evaluation of any willing individual in the screening process. YYEH were approached by RAs and asked if they were interested in learning more about the study. Interested youth received a short description of the study and tasks and were asked if they would like to see if they were eligible. After hearing about the study, if eligible and interested in participating, YYEH consented to participate and completed a baseline interview that assessed demographic, ABI, EF, and smoking-related domains. While the full study entails additional participation, this manuscript reports only on data collected at the baseline interview. Baseline interviews were completed in a quiet, private room within the drop-in center. Measures were administered by two trained RAs. The baseline interview took approximately an hour and a half to complete. Participants were given breaks and snacks as needed. Participants were compensated USD 25 for participation in the baseline interview. The study aimed to recruit 30 total YYEH, 15 with and 15 without a history of ABI. However, throughout the recruitment process, screenings identified very few individuals who did not have a history of ABI but met other inclusion criteria. The recruitment goal was adjusted to include thirty total participants regardless of ABI status, and twenty-three participants were successfully recruited. Three participants were excluded due to duplicate enrollment (e.g., signing up twice with different recruiters, providing a fake name) and incomplete data.

### 2.2. Measures

#### 2.2.1. Presence of Lifetime Acquired Brain Injury

To assess the presence of lifetime ABI, participants completed the Ohio State University TBI Identification Method (OSU TBI-ID+ABI) [13]. The OSU TBI-ID+ABI is the most widely used standardized screener for eliciting a person’s lifetime history of brain injury (from blunt force head trauma, shaking, strangulation, or overdose). The OSU TBI-ID+ABI is not intended to provide a diagnosis of ABI. Rather, it is intended to “estimate the likelihood that consequences have resulted from one’s lifetime exposure” [13]. The OSU TBI-ID+ABI is completed in approximately 3–5 min during a structured interview. The OSU TBI-ID+ABI has shown sound psychometric properties in adults [13] and those in the justice system [14]. Questions presented through the OSU TBI-ID+ABI are available in Appendix A. 

#### 2.2.2. Executive Functioning Measures

Executive functioning skills were measured using the Behavior Rating Inventory of Executive Function for Adults (BRIEF-A) [15,16,17]. The BRIEF-A is a normed, standardized questionnaire that produces two indices (i.e., Behavioral Rating Index (BRI) and Metacognition Index), which are combined to form a Global Executive Composite (GEC) score. The BRI is composed of four clinical subscales: inhibit, shift, emotional control, and self-monitor. The Metacognition Index is composed of five clinical subscales: initiate, working memory, plan/organize, task monitor, and organization of materials. Additionally, the BRIEF-A includes three validity scales (i.e., inconsistency, infrequency, and negativity). The BRIEF-A includes 75 items presented using a 3-point Likert scale, where participants rate how often a behavior is present (i.e., 1 = never, 2 = sometimes, 3 = often). Ratings are then translated into T-scores, where the mean score for each index is 50 and scores greater than or equal to65 indicate clinically significantly elevated levels of executive dysfunction. BRIEF-A administration was completed through the PARiConnect online portal using an iPad. While the BRIEF-A is a self-report measure, for the purposes of this study, research assistants read each question to participants to account for reading and literacy-related difficulties that may exist. The BRIEF-A takes approximately 10–15 min to complete. The BRIEF-A has demonstrated excellent internal consistency in adults [16,17] and those with TBI [18].

In addition to the BRIEF-A, the Dimensional Change Card Sort (DCCS) and Flanker Inhibitory Control and Attention (Flanker) subtests from the NIH Toolbox Cognitive Battery [19,20] assessed cognitive flexibility, inhibitory control, and selective attention. The NIH Toolbox Cognitive Battery has shown good reliability and has been validated for use with individuals with brain injury [19,20]. The NIH Toolbox Cognitive Battery program verbally presents instructions and takes approximately 10 min to complete on an iPad. The DCCS asks participants to match pictures based on either shape or color, with instructions varying throughout the assessment. The Flanker requires participants to focus their attention on a central stimulus while ignoring flanking stimuli. Both the DCCS and the Flanker test are scored based on accuracy and reaction time, where a higher score indicates better performance. An age-adjusted standardized score is provided by the NIH Toolbox following completion of the measures (mean = 100, standard deviation = 15). The NIH Toolbox has demonstrated acceptable internal consistency in adult populations [21].

### 2.3. Data Analysis

All participants were classified using two methods to allow for analyses focused on the number of reported lifetime injuries and, separately, the injury mechanism. Categorizations were determined based on responses to the first three questions of the Iowa Modification of the Ohio State University TBI Identification Method (OSU TBI-ID+ABI), which are shown in Appendix A. To determine if there were differences based on the number of reported lifetime injuries, participants were divided into one of three groups (i.e., no ABI, single, multiple). Those who reported a single loss of consciousness (LOC) from either blunt force trauma or loss of brain oxygenation were categorized into the single injury group. If participants reported multiple events of LOC from a drug overdose or being choked, or endorsed having a period of time where they experienced multiple repeated impacts to their head (not necessarily with a LOC), they were categorized into the multiple injury group. Participants who reported no LOC from either possible blunt force trauma or loss of oxygen and, additionally, did not report ever having a time in their life where they experienced multiple repeated impacts to their head were categorized in the no ABI group. The OSU TBI-ID+ABI collects data on the length of loss of consciousness, which may be useful when estimating the severity of a TBI. However, this method of categorization may not be appropriate for estimating the severity of hypoxic-anoxic injury (HAI; decrease or lack of oxygen to the brain). Therefore, differences based on injury severity were not evaluated.

To determine if there were differences based on the mechanism of injury, participants were divided into two groups (i.e., TBI only or HAI/Mixed). Participants were included in the TBI only group if they endorsed one or multiple experiences of blunt force trauma with no accounts of loss or complete disruption of oxygenation. Individuals were considered to have had an HAI if they endorsed loss of oxygen from either a drug overdose, choking, or potential strangulation. Participants who experienced an HAI injury independently or combined with one or multiple accounts of blunt force head trauma were categorized into the HAI/Mixed group.

All analyses were conducted using IBM SPSS Version 29 for Macintosh [22]. Descriptive statistics were generated for demographic (i.e., age, sex, ethnicity) and clinical variables (i.e., BRIEF-A and NIH Toolbox scores) both in the full sample as well as within each of the groups. Given the small number of participants with no history of ABI (n = 3), these individuals were characterized with descriptive statistics but were not included in the analytic sample for the remainder of the analysis. Researchers used nonparametric Mann–Whitney U tests to evaluate if there were significant differences in EF performance between those with single or multiple events of lifetime ABI. Similarly, Mann–Whitney U tests were also used to determine if there were significant differences in EF performance based on the mechanism of lifetime ABI (i.e., HAI/Mixed or TBI only). One participant chose not to participate in the DCCS subtest of the NIH Toolbox. Missing data are reported in the tables and accounted for in the statistical analysis.

## 3. Results

### 3.1. Participant Characteristics

Twenty-three individuals were included in the sample. On average, participants were 21.1 years old [range = 18 to 24 years]. Approximately half of participants identified as female (n = 12, 52.2%), and a majority were Black or African American (n = 14, 60.9%). The sample included in this study is roughly comparable to demographic samples of YYEH in developed countries, with non-White and LGBT youth being generally overrepresented compared to the total population [23]. A complete summary of demographic information is presented in Table 1.

### 3.2. OSU TBI-ID+ABI

Based on OSU TBI-ID+ABI reporting, 60.9% (n = 14) of YYEH in the sample lost consciousness when they sustained a TBI, and of these, 64.3% (n = 9) first lost consciousness prior to age 20. Over half (69.6%, n = 16) reported multiple ABIs throughout their lifetime. Finally, 47.8% of individuals endorsed loss of consciousness from either a drug overdose or from being purposefully choked. Eight participants (34.8%) reported exposure to other sources of possible brain injury including epilepsy or seizures, a stroke, brain tumor, brain edema, toxic effects of poisoning or by substances, infection (i.e., meningitis or encephalitis), brain bleed, child or adult maltreatment syndrome, or other loss of oxygen to the brain.

### 3.3. Executive Functioning

Descriptive statistics from the BRIEF-A and NIH Toolbox Cognitive Battery subtests are presented for the overall sample and for those who did not experience an ABI in Table 2. BRIEF-A scores for those in the overall sample and no ABI groups did not indicate clinically elevated scores, indicating EF within average limits.

### 3.4. Executive Functioning and Number of Injuries

EF scores were significantly higher (i.e., worse) for those with multiple injuries compared to those with a single injury on the following clinical scales of the BRIEF-A: shift, self-monitor, task monitor, and GEC (all *p* ≤ 0.05), as presented in Table 3. The associated Hodges–Lehmann estimates of the location shift in the BRIEF-A scores from single to multiple injuries were 13, 17.5, 14, and 15 for the shift, self-monitor, task monitor, and GEC scales, respectively. These effect sizes estimate the median difference between scores from the two groups (multiple injury–single injury). For example, for the shift scale, the median difference when comparing participants with multiple injuries to those with a single injury was 13, indicating that generally the scores for the participants with multiple injuries were 13 points higher than those with a single injury. These higher scores on the BRIEF-A indicate that those with multiple injuries have poorer EF compared to those with a single injury. No significant differences between groups were found for the NIH Toolbox subscales.

### 3.5. Executive Functioning and Injury Mechanism

There were no significant between-group differences for either the BRIEF-A or the NIH Toolbox measures based on the mechanism of lifetime ABI (Table 3).

## 4. Discussion

The purpose of this pilot study was to address a critical gap in research by collecting information on the lifetime history of ABI and the current level of EF among a small sample of YYEH. We evaluated differences in EF based on the number (i.e., single or multiple) and mechanism (i.e., TBI or HAI/mixed) of lifetime ABI. Our hypotheses were partially supported. Our results provide preliminary data suggesting that there is a high likelihood that YYEH may show high rates of multiple instances of ABI, and that many of those include HAI, not just TBI. Additionally, in this sample, those who have experienced multiple ABIs showed poorer EF than those with a history of one ABI. Participants with a history of multiple ABIs had significantly higher scores on multiple clinical scales of the BRIEF-A, indicating poorer EF. Our findings are similar to previous studies investigating the impact of multiple traumas on functional and mental health outcomes. In a meta-analysis conducted by Op den Kelder et al. [24], the authors reported that youth aged 0–25 who had experienced a trauma performed worse on measures of EF than those with no trauma exposure. Surprisingly, we did not find clinically concerning EF scores in our overall YYEH sample. This may be explained by our small sample size and by our use of one recruitment site, which does not account for YYEH who are not accessing housing resources at another location or at all. More specifically, YYEH who were approached for this study were recruited within a drop-in center, indicating that they had taken the initiative to seek some level of support independently. It is possible that individuals who seek support from a drop-in center are utilizing or exercising EFs in a different way than youth who do not seek such services. For example, youth who wish to access a drop-in center for the first time must exercise a combination of EFs to find the location of the center, plan the route, identify and utilize transportation, and then must show initiation while at the center to access the services provided once inside (i.e., medical care, employment resources). Future studies may consider incorporation of multiple sites for recruitment across cities and potential state lines to increase generalizability. Additionally, studies may consider including participants from not only drop-in centers but also from street-recruited samples to further identify needs as they relate to supports for EF.

This pilot study illustrates the increased likelihood for YYEH to sustain not just TBI but also brain injury from HAI, such as strangulation and drug overdose. We did not find evidence of significant differences in EF measures between groups based on the mechanism of ABI. However, it is likely that the small sample size limited our ability to detect differences between groups. In addition, it is possible that underlying conditions such as ADHD, mechanism or severity of injury, or other premorbid mental health conditions may be present in the population and were not accounted for in this analysis. Recent research identifies the intersectionality of prior TBI with psychological trauma, and that each independently may predict later difficulties [25]. Future studies must continue to explore these complex relationships in vulnerable groups such as YYEH to provide opportunities for improved interventions and service access. It is also possible that more noticeable differences may be detected in acute phases of recovery, though evaluating this presents a challenge in populations of individuals vulnerable to social and health disparities [26,27]. Research evaluating outcome differences based on the mechanism of ABI is limited, though studies suggest that patients with non-traumatic brain injury may have worse outcomes [28]. In the current pilot study, we did not collect information on health care received following ABI events. Given the challenges that YYEH may face in accessing health care, this is an important consideration for future work.

During the recruitment process, we found it difficult to identify YYEH who met our other inclusion criteria and had not experienced an ABI throughout their lifetime, limiting us to recruiting only three YYEH with no history of ABI. This may have been due to the eligibility criteria of the QUEST study, which required that YYEH be current smokers who wanted to stop smoking. However, our inability to recruit tobacco-using YYEH without a lifetime history of ABI may be indicative of a different phenomenon. Many YYEH report experiences of trauma from home or from street victimization after leaving home [29]. The location (i.e., home vs. street) where trauma was experienced is related to later PTSD or substance abuse disorders, including tobacco abuse [29]. Given that previous research has shown exposure to head trauma precedes tobacco use for many YYEH [6], it is possible that the traumatic experiences resulting in ABI are the same that lead to tobacco use. Therefore, determining where YYEH experienced ABI may be an important consideration for determining how trauma, nicotine use, and EF are interrelated.

### Study Limitations

Our study faced several limitations. We faced challenges during recruitment that prevented us from recruiting our target of 30 individuals. While this was an exploratory pilot study, we recognize that our sample size was underpowered, further limiting our analysis and the interpretation of findings presented in the manuscript. As a preliminary investigation, it does provide new information about the types of ABI experienced by YYEH accessing services at a drop-in center and is the first to evaluate the EF abilities of these same youth. Results from this study can be used to encourage future work focusing on vulnerable and under-resourced populations.

We were only able to recruit three individuals with no history of ABI and chose not to include this group in our analysis. This may suggest that the lifetime presence of ABI in YYEH is much higher than previously reported, and continued research is needed to further determine how many YYEH have a lifetime history of ABI. As mentioned, we must consider the potential limitations from the inclusion criteria of the QUEST study, specifically the requirement that participants were current tobacco users who were also interested in quitting. This may limit the generalizability of these findings. Given previous literature showing that chronic tobacco use negatively impacts EF [30], it is impossible to make conclusive statements about the impact of ABI on EF in this population without considering that there may also be a compounded effect of nicotine addiction on EF performance. We were also limited in our ability to capture the lifetime history of ABI. Though we used a gold standard in TBI identification, it was difficult to clearly account for the mechanism of multiple injuries, how many resulted in loss of consciousness, and whether YYEH sought medical treatment after head trauma. These limitations made it difficult to clearly delineate between the number of brain injury exposures and type. Additionally, we were limited in our ability to categorize participants based on injury severity. This study evaluated the estimated lifetime presence of ABI, including TBI. Given that methods of determining severity for an ABI may not be equivocal to methods for determining severity of a TBI, we were unable to categorize participants based on injury severity. Our choice to use only the first three questions on the OSU TBI-ID+ABI may have limited our ability to detect individuals with multiple injuries from other causes. Because YYEH are at a high risk for inter-personal violence and assault, including strangulation, future research should specifically ask about these types of ABI exposures.

## 5. Conclusions

This pilot study presents important preliminary data and is the first to explore the intersection of EF abilities and lifetime history of ABI in YYEH. YYEH represent an understudied population that is vulnerable to EF impairments from chronic tobacco use, ABI, and other means. This population is at a particularly high risk of multiple ABIs due to increased exposure to dangerous situations and increased risk for a prior history of mental health diagnoses that predispose individuals to ABI. To best serve YYEH, it is essential to consider how interventions can be adapted to account for both the possibility of ABI and potential EF difficulties. For example, individuals working with this population in drop-in locations may offer assistance for youth when completing important documentation such as medical forms and housing or job applications. Staff may provide quiet spaces for youth to access to complete activities without external stimuli. Considerations should be given to the varying presentation of ABI exposure (i.e., traumatic vs. non-traumatic) as well as how a history of ABI impacts the ability to successfully access services. To promote access to services and supports, providers must understand the potential barriers posed by EF difficulties and past ABIs among YYEH. Future research may consider further exploring the impacts of gender and social support on the EF differences in YYEH. By developing this foundational understanding, providers can implement accommodations that promote equitable access to essential resources for this population.

## Figures and Tables

**Table 1:** Participant demographic information.

	Total Sample	No ABI	Number of Injuries		Injury Mechanism	
	(n = 23)	(n = 3)	Single (n = 4)	Multiple (n = 16)	TBI Only (n = 9)	HAI/Mixed (n = 11)
Age						
Mean	21.13	22.00	20.75	21.06	20.78	21.18
SD	1.66	1.00	0.96	1.88	1.09	2.14
Age at first ABI	(n = 14)		(n = 4)	(n = 10)	(n = 6)	(n = 9)
Mean	16.79	N/A	16.25	17.00	16.67	16.88
SD	3.53	N/A	4.50	3.33	3.88	3.52
	n (%)	n (%)	n (%)	n (%)	n (%)	n (%)
Gender ^a^						
Male	11 (47.83)	3 (100)	2 (50.00)	6 (37.50)	5 (55.56)	3 (27.27)
Female	12 (52.17)	0 (0)	2 (50.00)	10 (62.50)	4 (44.44)	8 (72.73)
Sexual Orientation						
Bisexual	5 (21.74)	0 (0)	0 (0)	5 (31.25)	1 (11.11)	4 (36.36)
Gay or Lesbian	1 (4.35)	0 (0)	0 (0)	1 (6.25)	0 (0)	1 (9.09)
Heterosexual	13 (56.52)	2 (66.67)	4 (100)	7 (43.75)	7 (77.78)	4 (36.36)
Self-Describe	3 (13.04)	1 (33.33)	0 (0)	2 (12.50)	1 (11.11)	1 (9.09)
Prefer not to answer	1 (4.35)	0 (0)	0 (0)	1 (6.25)	0 (0)	1 (9.09)
Race						
American Indian or Alaska Native	1 (4.35)	0 (0)	0 (0)	1 (6.25)	0 (0)	1 (9.09)
Black or African American	14 (60.87)	3 (100)	3 (75.00)	8 (50.00)	7 (77.78)	4 (36.36)
Native Hawaiian Pacific Islander	1 (4.35)	0 (0)	1 (25.00)	0 (0)	1 (11.11)	0 (0)
White	4 (17.39)	0 (0)	0 (0)	4 (25.00)	1 (11.11)	3 (27.27)
Multiracial	2 (8.70)	0 (0)	0 (0)	2 (12.50)	0 (0)	2 (18.18)
Prefer not to answer	1 (4.35)	0 (0)	0 (0)	1 (6.25)	0 (0)	1 (9.09)
Length of Loss of Consciousness						
None	9 (39.13)	3 (100)	0 (0)	6 (37.50)	3 (33.33)	3 (27.27)
<30 min	11 (47.83)	0 (0)	3 (75.00)	8 (50.00)	5 (55.56)	6 (54.55)
30 min–24 h	2 (8.70)	0 (0)	0 (0)	2 (12.25)	0 (0)	2 (18.18)
>24 h	1 (4.35)	0 (0)	1 (25.00)	0 (0)	1 (11.11)	0 (0)

Note. Individuals may experience loss of consciousness when they sustain an acquired brain injury; however, the lack of loss of consciousness does not always indicate that no brain injury occurred. ABI = Acquired Brain Injury; TBI = Traumatic Brain Injury; HAI = Hypoxic-Anoxic Injury. ^a^ Self-reported gender and biological sex did not differ in this sample.

**Table 2:** Median and IQR/range of BRIEF-A and NIH Toolkit for the total sample and the no ABI group.

	Total Sample (n = 23)	No ABI (n = 3)
BRIEF-A	Median [IQR]	Median [Range]
Inhibit	63 [53–74]	57 [53–63]
Shift	60 [47–64]	56 [39–64]
Emotional Control	54 [49–69]	43 [40–49]
Self-Monitor	59 [50–76]	59 [46–72]
BRI	62 [51–70]	53 [46–58]
Initiate	56 [47–63]	56 [53–60]
Working Memory	59 [53–73]	53 [49–66]
Plan Organize	57 [46–68]	52 [52–57]
Task Monitor	54 [45–63]	45 [45–54]
Organization of Materials	50 [45–56]	47 [45–47]
Metacognition	56 [48–66]	52 [50–56]
GEC	61 [50–70]	53 [48–58]
NIH Toolbox		
DCCS	87 [83–94] ^a^	78 [76–101]
Flanker	84 [72–97]	89 [69–99]

Note. One participant chose not to participate in the DCCS subtest of the NIH Toolbox. Affected values are indicated. ABI = Acquired Brain Injury; BRI = Behavior Rating Index; GEC = Global Executive Composite; DCCS = Dimensional Change Card Sort; Flanker = Flanker Inhibitory Control and Attention. ^a^ n = 22.

**Table 3:** Median and IQR of BRIEF-A and NIH Toolkit by group and Mann–Whitney U test results.

	Number of Injuries (n = 20)			Injury Mechanism (n = 20)		
	Single (n = 4)	Multiple (n = 16)	*p*	TBI Only (n = 9)	HAI/Mixed (n = 11)	*p*
BRIEF-A	Median [IQR]	Median [IQR]		Median [IQR]	Median [IQR]	
Inhibit	58.50 [47–67]	65 [54–76.25]	0.25	67 [48–80.50]	63 [53–74]	0.94
Shift	47 [41–53.75]	60 [49.25–69]	0.04 *	47 [45–66.50]	60 [56–69]	0.20
Emotional Control	51.50 [44.50–65.25]	62.50 [54–71.25]	0.21	54 [48–70.50]	60 [54–67]	0.84
Self-Monitor	50 [47–53]	67.50 [52.25–79]	0.02 *	54 [48–78]	63 [59–76]	0.23
BRI	51.50 [42.25–63]	67 [54–76.50]	0.06	54 [48.50–79.50]	67 [57–70]	0.41
Initiate	43 [40.75–55.75]	61.50 [47.75–67.50]	0.08	47 [41.50–68]	63 [53–63]	0.20
Working Memory	56 [47.75–59]	69 [56–73]	0.12	59 [49.50–76]	69 [56–73]	0.60
Plan Organize	46.50 [41.75–57.25]	65 [48–77.50]	0.08	49 [44–70.50]	65 [54–78]	0.30
Task Monitor	45 [41.25–48.75]	59 [50–71]	0.02 *	50 [45–65.50]	59 [50–68]	0.20
Organization of Materials	47.50 [42.75–52.25]	56 [47.75–58]	0.18	53 [43.50–59.50]	53 [47–56]	0.82
Metacognition	46.50 [44.25–54]	63.50 [53–69.75]	0.08	52 [44.50–69.50]	63 [56–69]	0.37
GEC	49 [44.75–58.50]	66 [51.25–71]	0.05 *	51 [46–75.50]	64 [55–70]	0.37
NIH Toolbox						
DCCS	87 [83–99] ^a,b^	87 [83.25–92.75]	0.88	87 [80.75–92.75] ^c^	87 [84–94]	0.72
Flanker	96 [86.75–98.50]	75 [69.75–95.50]	0.15	81 [71.50–96]	92 [72–101]	0.66

Note. One participant chose not to participate in the DCCS subtest of the NIH Toolbox. Affected values are indicated. TBI = Traumatic Brain Injury; HAI = Hypoxic–Anoxic Injury; BRI = Behavior Rating Index; GEC = Global Executive Composite; DCCS = Dimensional Change Card Sort; Flanker = Flanker Inhibitory Control and Attention. ^a^ n = 3. ^b^ = range reported. ^c^ n = 8. * *p* < 0.05.

## Data Availability

Data will be made available by the corresponding author upon reasonable request.

## Abbreviations

The following abbreviations are used in this manuscript:

YYEH	Youth and young adults experiencing homelessness
EF	Executive functioning
ABI	Acquired brain injury
ACE	Adverse childhood event
QUEST	Quitting Using Executive Strategy Training
OSU TBI-ID+ABI	The Ohio State University Traumatic Brain Injury Identification Method
BRIEF	Behavior Rating Inventory of Executive Functioning
GEC	Global executive composite
BRI	Behavioral rating index
DCCS	Dimensional change card sort
LOC	Loss of consciousness
TBI	Traumatic brain injury
HAI	Hypoxic/anoxic injury

## Appendix A

OSU TBI-ID+ABI Questions (see [13])

Q1. Please think about injuries you have had during your entire lifetime, especially those that affected your head or neck. It might help to remember times you went to the hospital or emergency department. Think about injuries you may have received from a car or motorcycle wreck, bicycle crash, being hit by someone, playing sports, or an injury during military service. 1a. Thinking about any injuries you have had in your lifetime, were you ever knocked out, or did you lose consciousness? 1b. What was the longest time you were knocked out or unconscious? 1c. How old were you the first time you were knocked out or lost consciousness? Q2. Have you ever had a period of time in which you experienced multiple, repeated impacts to your head (e.g., history of abuse, contact sports, military duty)? Q3. Have you ever lost consciousness from a drug overdose or being choked?
